# Gut Bacterial Community Structure and Function Prediction of *Lygus pratensis* at Different Developmental Stages

**DOI:** 10.3390/insects17020168

**Published:** 2026-02-03

**Authors:** Tailong Li, Pengfei Li, Mengchun Li, Kunyan Wang, Changqing Gou, Hongzu Feng

**Affiliations:** 1 Agricultural College, Tarim University, Alar 843300, China; 10757242048@stumail.taru.edu.cn (T.L.); 10757232030@stumail.taru.edu.cn (K.W.); 2 Key Laboratory of Integrated Pest Management (IPM), Xinjiang Production and Construction Corps in Southern Xinjiang, Tarim University, Alar 843300, China; 10757202064@stumail.taru.edu.cn; 3 College of Life Science and Technology, Tarim University, Alar 843300, China; 4 Nanyang Academy of Agricultural Sciences, Nanyang 473000, China; limech75@163.com

**Keywords:** mirid bug, gut bacteria, microbial community structure, high-throughput sequencing

## Abstract

*L. pratensis* is a significant pest of cotton. Clarifying the intestinal bacterial structure of *L. pratensis* can provide a theoretical basis for the development of new pest biological control strategies. In this study, high-throughput sequencing was employed to characterize the intestinal bacterial communities across five *L. pratensis* populations, and the functions of their core metabolic pathways were predicted. The results showed that the intestinal bacterial communities of the five *L. pratensis* populations comprised 16 phyla, 25 classes, 54 orders, 85 families, 133 genera, and 187 species. Diversity analysis revealed that the diversity of the intestinal bacterial community exhibited a dynamic trend of first increasing and then decreasing during the pest’s growth and development. Specifically, the Shannon and Simpson diversity indices of the nymphal stage were significantly higher than those of the egg and adult stages (*p* < 0.05). The dominant phylum, class, order, family, genus and species shared by the five groups were Proteobacteria (93.17%), Gammaproteobacteria (48.71%), Rickettsiales (43.83%), Anaplasmataceae (49.39%), *Wolbachia* (43.83%) and *Wolbachia* (43.82%). Among them, *Acinetobacter* was mainly found in the first instar nymph stage, and *Serratia* was mainly distributed in the fifth instar nymph and female and male adults. Functional prediction results showed that the intestinal bacterial community was mainly enriched in core pathways, including metabolism, genetic information processing, and environmental information processing. This study provides a new target for green prevention and control of *L. pratensis* and also provides a theoretical basis for further elucidating the succession law and functional mechanism of its gut microbiota.

## Simple Summary

In this study, Illumina MiSeq high-throughput sequencing combined with PICRUSt2 functional prediction was used to analyze the intestinal characteristics of five populations of *L. pratensis*: eggs, nymphs (first and fifth instars), and adults (females and males). The results demonstrated significant differences in the diversity of intestinal bacterial communities among the five populations, with diversity showing a dynamic pattern of first increasing and then decreasing as the instar advanced. Specifically, the bacterial community diversity at the nymphal stage was significantly higher than that at the egg and adult stages. Across all populations, the intestinal bacteria were dominated by the phylum Proteobacteria and the genus *Wolbachia*. Functional prediction of the intestinal bacteria revealed that their functions were mainly concentrated in pathways such as metabolism and genetic information processing. This study lays a theoretical foundation for further elucidating the succession dynamics and functional mechanisms of the intestinal bacterial community in *L. pratensis*.

## 1. Introduction

In insect life activities, gut microbiota play an irreplaceable role. They actively participate in various physiological processes such as nutrient digestion, material metabolism, immune defense, reproductive development, and environmental adaptation of the host [1,2]. Xiang et al. [3] showed that the intestinal tract of insects is a selective colonization environment for microorganisms. The structure of insect flora with different feeding habits is specific. Microorganisms enter in three ways: food intake, maternal vertical transmission, and environmental-level acquisition. Numerous studies have shown that, due to the influence of various factors, the gut bacteria of insects across individuals exhibit significant differences in community structure and function [4]. For example, in phytophagous insects, gut microbes can secrete a variety of digestive enzymes to participate in food digestion, synthesize essential amino acids and vitamins, and enhance the insects’ adaptability to plant hosts [5]. For example, the gut microbiome of *Helicoverpa armigera* (Lepidoptera: Noctuidae) larvae is mainly composed of cellulose-degrading bacteria. The gut microbiome in the adult stage changes significantly compared with the larval stage, and the relative abundance of energy metabolism-related microorganisms increases [6]. Xia et al. [7] found that the intestinal microbial diversity of different instar nymphs of *Plutella xylostella* (Lepidoptera: Plutellidae) was different. The diversity of bacteria in the larval stage was the highest. The first instar larvae were rich in Ruminococcus and Prevotella, and the fourth instar larvae were rich in Marseilla and Comamonas. The diversity of the pupal stage was the lowest. Lu et al. [8] showed that the diversity of gut microbiota of *Culex pipiens pallens* (Diptera: Culicidae) pallens was the highest in the pupal stage and the lowest in the adult stage. The dominant flora was mainly Bacteroides. There were significant differences in the composition of the gut microbiota across developmental stages and functional differentiation. Therefore, analyzing the community dynamics and functional characteristics of gut microbiota at different developmental stages of pests can provide important insights into the mechanisms of microbial–host interactions and into the development of new pest control technologies.

*Lygus pratensis* (Linn, 1758) (Heteroptera: Miridae) is a dominant species and polyphagous pest of Miridae in cotton fields in Xinjiang [9]. Ingestion of host plant juice by piercing-sucking mouthparts leads to leaf curling, flower and boll falling, and fruit deformity, which seriously affects the yield and quality of crops [10]. In recent years, due to the wide-spread planting of transgenic *Bacillus thuringiensis* (Bt) cotton in Xinjiang, Bt cotton accounted for 95% of the total area of cotton planting in China by 2018 [11]. As well as the combined effects of changes in planting patterns, structural adjustment of the planting industry, and changes in crop layout, the occurrence area and number of *L. pratensis* in the southern cotton area of Xinjiang have increased year by year. It has become the primary pest in local cotton areas, and its damage is expected to continue to expand [12,13,14].

At present, the research on *L. pratensis* mainly focuses on biological characteristics, occurrence regularity, resistance monitoring, and chemical control, but the research on its gut bacteria has not been reported. The life history of *L. pratensis* includes three stages: egg, nymph (5 instars), and adult. There are significant differences in feeding habits, physiological status, and habitat conditions across developmental stages [15]. It is speculated that the gut bacteria community may also change accordingly. In this study, high-throughput sequencing was used to systematically analyze the community composition, diversity, and structural differences in gut microbes across the developmental stages of eggs, nymphs, and adults of *L. pratensis*, and to reveal the potential physiological functions of gut microbes through functional prediction. The purpose of this study was to clarify the relationship between gut microbiota and the growth and development of *L. pratensis*, and to provide a theoretical basis for the development of green prevention and control technology of *L. pratensis* based on gut microbiota.

## 2. Materials and Methods

### 2.1. Test Insect Source

From June to September 2025, during the peak season for *L. pratensis* infestations, sample collection was conducted at the Experimental Base of the Agricultural Science Research Institute of the First Division of the Xinjiang Production and Construction Corps (81°23′25″ E, 40°32′8″ N). Insects were collected from cotton fields (planted in a one-film, four-rows pattern with 10 cm plant spacing and (66 + 10) cm row spacing, yielding a density of 150,000–180,000 plants/ha) and adjacent weeds. No pesticides were applied to the cotton fields or surrounding areas during the entire growth period. The collected insects (nymphs and adults) were fed separately with sterile water under suitable conditions. The feeding conditions were as follows: temperature (25 ± 1) °C, relative humidity (70 ± 5)%, photoperiod 15L:9D, and light intensity 480 ± 20 lux.

### 2.2. Total DNA Extraction and PCR Amplification

After 24 h of gut clearance, uniformly developed healthy *L. pratensis* at egg, 1st/5th instar nymph, and female/male adult stages were sampled (3 biological replicates per stage; 10 individuals/replicate; and 300 eggs/replicate for egg stage). Samples were surface-sterilized with 75% ethanol (30 s) and triple-rinsed with sterile saline. Aseptic gut dissection was performed on a clean bench; complete intestines were excised, stripped of adhering fat, body, and tissues, and suspended in 1 mL 0.9% sterile saline. Eggs were directly added to 1 mL 0.9% sterile saline in centrifuge tubes. All samples were homogenized to prepare microbial suspensions for DNA extraction.

### 2.3. Intestinal Sample Preparation

Genomic DNA was extracted using the TGuide S96 Magnetic Soil/Stool DNA Kit (Tiangen Biotech, Beijing, China) according to the manufacturer’s protocol. DNA quality was verified by 1.8% agarose gel electrophoresis, and concentration/purity were determined using a NanoDrop 2000 UV-Vis spectrophotometer (Thermo Scientific, Wilmington, DE, USA). The V3–V4 hypervariable region of the bacterial 16S rRNA gene was amplified with indexed primers 338F (5′-ACTCCTACGGGAGGCAGCA-3′) and 806R (5′-GGACTACHVGGGTWTCTAAT-3′). PCR was performed in a 20 μL reaction system containing 5–50 ng DNA template, 0.3 μL each of 10 μM forward/reverse primers, 5 μL KOD FX Neo Buffer, 2 μL 2 mM dNTPs, 0.2 μL KOD FX Neo, and ddH_2_O to volume. The PCR program included an initial denaturation at 95 °C for 5 min, followed by 20 cycles of 95 °C (30 s), 50 °C (30 s), and 72 °C (40 s), and a final extension at 72 °C for 7 min. Amplicons were purified using the Omega DNA Purification Kit (Omega Inc., Norcross, GA, USA) and quantified using the Qsep-400 (BiOptic, New Taipei City, Taiwan, China). Paired-end (2 × 250) sequencing of the amplicon library was conducted on an Illumina Novaseq 6000 platform (Beijing Biomarker Technologies, Beijing, China).

### 2.4. Sequencing Data Processing and Analysis

Using FLASH v1.2.11 software, paired-end reads were merged to obtain raw sequences [16]. Quality filtering was conducted to generate clean reads, and chimeric sequences were removed to obtain effective reads for downstream analysis. The DADA2 plugin implemented in QllME 2 [17,18] was used fordenoising, dereplication, chimera removal, and construction of Amplicon Sequence Variants (ASVs) and feature tables [19]. Taxonomic assignment was performed based on the Silva 138 database, and QllME 2 was used to calculate the relative abundance of bacterial taxa.

Alpha diversity indices (ACE, Chao1, Shannon, and Simpson) and beta diversity distance matrices (Bray–Curtis and unweighted UniFrac) were calculated within QIIME 2 for subsequent statistical analyses.

Functional profiles of the intestinal bacterial communities were predicted using PICRUSt2 v2.3.0 [20] based on ASV data.

### 2.5. Statistical Analysis

All statistical analyses were performed to evaluate differences in intestinal bacterial diversity, community structure, and predicted functions among different developmental stages of *L. pratensis*. Prior to parametric analyses, the normality and homogeneity of variance of alpha diversity indices were assessed using the Shapiro–Wilk test and Levene’s test, respectively. When data did not meet the assumptions of parametric tests, non-parametric methods were applied.

Multiple comparisons among developmental stages were adjusted using the Benjamini–Hochberg false discovery rate (FDR) correction to control for type I errors.

Non-metric multidimensional scaling (NMDS) based on Bray–Curtis and unweighted UniFrac distance matrices was used to visualize differences in microbial community composition. Permutational multivariate analysis of variance (PERMANOVA) was performed using the adonis function with 999 permutations to test the significance of community differences among groups.

All statistical analyses and graphical visualizations were conducted using R software (version 4.0.3), and statistical significance was defined as *p* < 0.05 unless otherwise stated.

## 3. Results

### 3.1. Annotation and Evaluation of Bacterial Species in the Gut of L. pratensis

**Figure 1:**
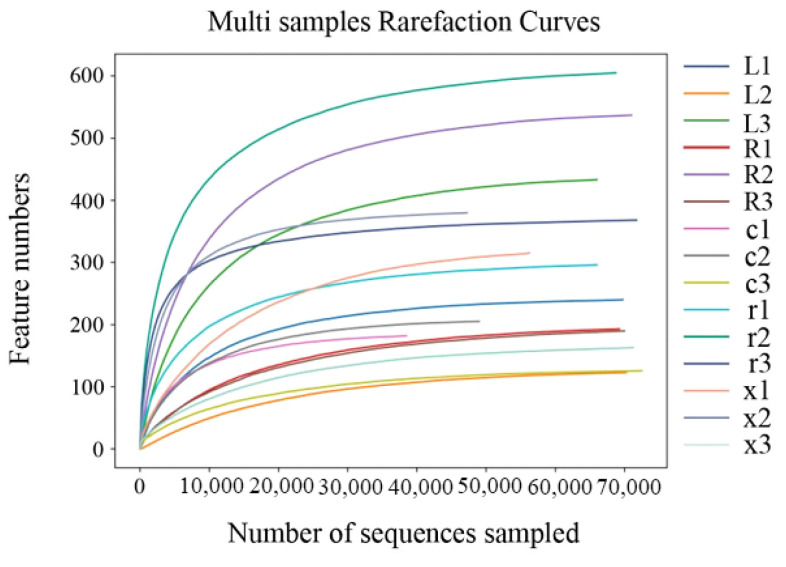
Rarefaction curves of gut bacterial composition in *L*. *pratensis* at different developmental stages.

The Illumina MiSeq platform was used to sequence the V3–V4 region of the 16S rDNA gene of the gut bacteria of the five developmental stages of eggs, first and fifth instar nymphs, and female and male adults, with three biological replicates in each stage. After quality control of the original sequence, 976,911 high-quality sequences were obtained (average 65,127 per sample); after denoising, splicing, and chimera removal, 960,615 sequences were obtained (Table 1). A total of 3378 ASVs were obtained from 15 samples by clustering at 97% sequence similarity. Among them, 19 ASVs were co-owned by the gut bacteria of different developmental stages of *L. pratensis*. As shown in Figure 1, the Sobs index of intestinal bacteria in *L. pratensis* across different developmental stages gradually stabilized, indicating that the sequencing data in this study fully cover the community’s species diversity and meet the needs of subsequent analysis.

### 3.2. The Main Intestinal Bacterial Community Structure of L. pratensis

**Figure 2:**
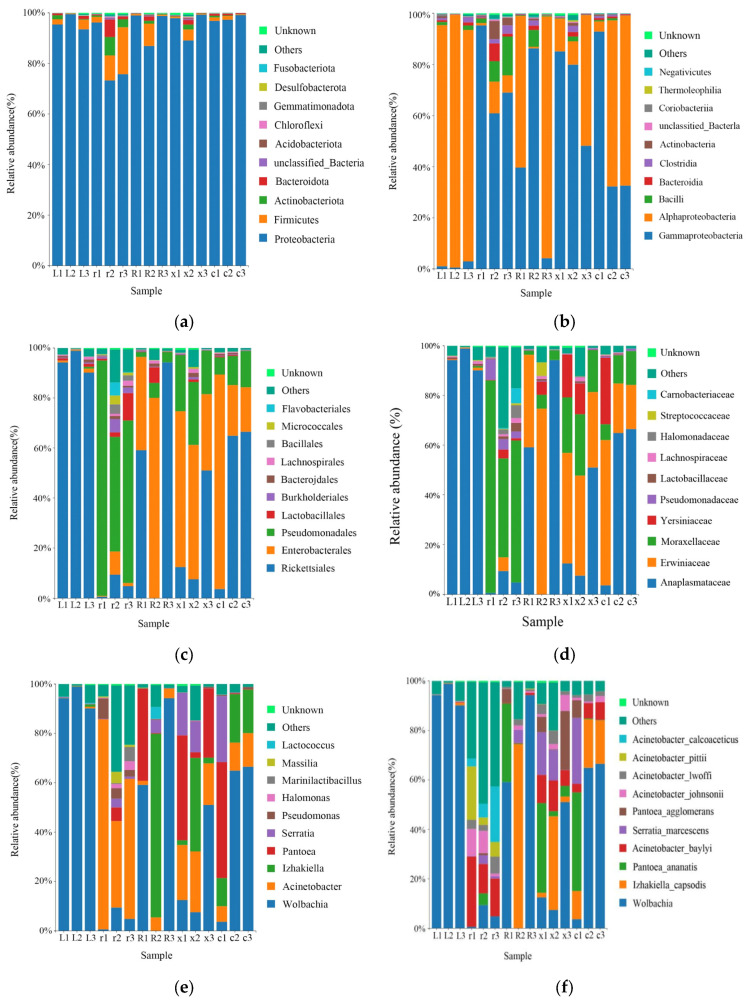
Relative abundance of the top 10 gut bacteria at the phylum, class, order, family, genus, and species levels in different developmental stages of the L. pratensis: (**a**) relative abundance at the phylum level; (**b**) relative abundance at the class level; (**c**) relative abundance at the order level; (**d**) relative abundance at the family level; (**e**) relative abundance at the genus level; and (**f**) relative abundance at the species level.

A total of 16 phyla, 25 classes, 54 orders, 85 families, 133 genera, and 187 species of bacteria were obtained by taxonomic annotation of the characteristic sequences. At the phylum level, the main bacteria were Proteobacteria (93.17%), Firmicutes (3.66%), Actinobacteria (1.19%), and Bacteroidota (1.10%). At the class level, the main classes were Gammaproteobacteria (48.71%), Alphaproteobacteria (44.46%), Bacillus (2.59%), Bacteroidia (1.10%), Clostridia (1.02%), and Actinobacteria (0.99%). At the order level, the main order were Rickettsiales (43.83%), Enterobacterales (26.74%), Pseudomonadales (21.06%), Lactobacillales (1.61%), Burkholderiales (0.81%), and Bacteroidales (0.60%). At the family level, the main families were *Anaplasmataceae* (43.83%), *Erwiniaceae* (22.01%), *Moraxellaceae* (19.30%), *Yersiniaceae* (4.47%), *Pseudomonadaceae* (1.22%), *Lactobacillaceae* (0.59%), *Lachnospiraceae* (0.58%), and so on. At the genus level, *Wolbachia* (43.83%), *Acinetobacter* (18.92%), *Izhakiella* (11.09%), *Pantoea* (10.91%), *Serratia* (4.47%), *Pseudomonas* (1.22%), and so on, were the main genera. At the species level, the main species were *Wolbachia* (43.82%), *Izhakiella_capsodis* (11.09%), *Pantoea_ananatis* (8.00%), *Acinetobacter_baylyi* (6.95%), *Serratia_marcescens* (4.46%), *Pantoea_agglomerans* (2.91%), *Acinetobacter_johnsonii* (2.51%), *Acinetobacter_lwoffii* (2.25%), *Acinetobacter_pittii* (2.08%) and *Acinetobacter_calcoaceticus* (2.07%) (Figure 2).

### 3.3. The Results of Intestinal Bacterial Diversity Analysis at Different Developmental Stages of L. pratensis

#### 3.3.1. Alpha Diversity Analysis of Intestinal Bacteria

**Figure 3:**
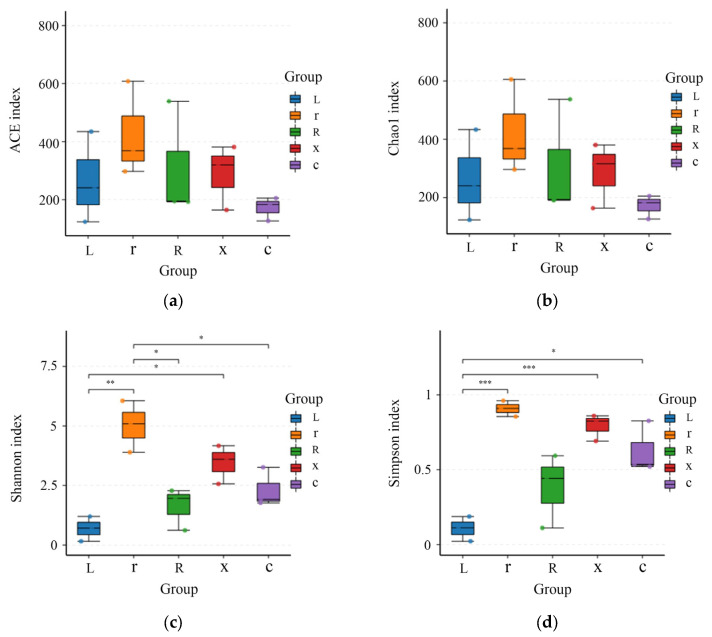
Analysis of gut bacterial alpha diversity at different developmental stages of the *L. pratensis*: (**a**) ACE index: Abundance-based coverage estimates are indices used to estimate the number of species in a community; (**b**) Chao1 index: One of the measures of species richness; (**c**) Shannon index: Used to estimate one of the microbial diversity indices in the sample; and (**d**) Simpson index: Used to estimate one of the microbial diversity indices in the sample. The abscissa is the group name, and the ordinate is the corresponding alpha diversity index. In the box line diagram, the meaning of each symbol is as follows: the upper and lower end lines of the box: the upper and lower quartile range (IQR); median line: median; upper and lower edges: maximum and minimum peripheral value (1.5 times IQR); and points outside the upper and lower edges: represents an outlier. The number on the line between the columns is the *p* value of the *t* test (if the *p* value > 0.05, the *p* value is not displayed by default). * indicates a significant difference (*p* < 0.05), ** indicates a highly significant difference (*p* < 0.01), *** indicates an extremely significant difference (*p* < 0.001).

Alpha diversity indices can reflect the diversity of a sample’s flora. In this study, Simpson, Shannon, Chao1, and ACE were selected to analyze the diversity of gut bacteria in different developmental stages of *L. pratensis*. The smaller the Simpson index, the higher the Shannon index, indicating that greater species diversity in the sample corresponds to larger Chao1 and ACE index values and higher community richness. As shown in Figure 3, the intestinal bacterial richness indices (ACE and Chao1) decreased from young nymphs to old nymphs and adults. However, there was no significant difference (*p* > 0.05), indicating that the bacterial richness levels among the samples were similar. The Simpson diversity index (Simpson index and Shannon index) gradually increased. The Simpson index showed that there were differences between eggs and female adults (*p* < 0.05), and the Simpson index of the first instar nymphs and male adults was significantly higher than that of eggs (*p* < 0.001). The Shannon index of eggs was significantly lower than that of the first instar nymphs (*p* < 0.01) and significantly lower than that of male adults. The Shannon index of first instar nymphs was significantly higher than that of fifth instar nymphs and female adults.

#### 3.3.2. Beta Diversity Analysis of Intestinal Bacteria

**Figure 4:**
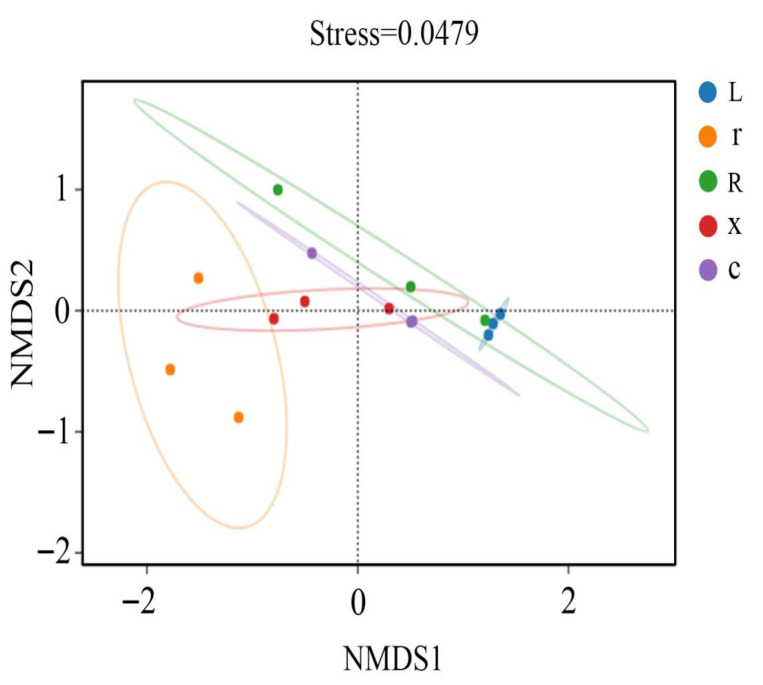
NMDS analysis of gut bacterial beta diversity at different developmental stages of *L. pratensis*. Note: Each point in the figure represents a sample; different colors represent different groups; and the oval circle indicates that it is a 95% confidence ellipse (that is, if there are 100 samples in the sample group, 95 of them will fall into it). When Stress is less than 0.1, it can be considered a good sort. When the Stress is less than 0.05, it is well-represented. It is generally believed that when Stress is less than 0.2, NMDS analysis is reliable. The closer the sample is to the coordinate diagram, the higher the similarity.

**Figure 5:**
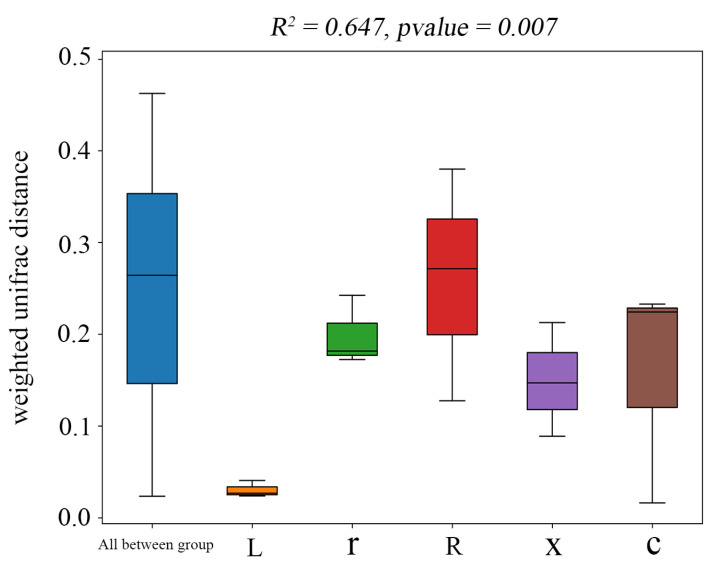
PERMANOVA of gut bacterial communities at different developmental stages of *L. pratensis*. Note: Sitting indicates Beta distance; the box plot above “All between group” represents the Beta distance data of samples between all groups; and the following box plots are the Beta distance data between samples in different groups. The R^2^ obtained by PERMANOVA analysis indicates the degree of interpretation of the difference between different groups of samples, that is, the ratio of group variance to total variance. The greater the R^2^, the higher the degree of interpretation of the difference between the groups, and the greater the difference between the groups. When the *p*-value is less than 0.05, the test is reliable.

In this study, at the ASV classification level, the diversity of microbial communities across different samples was systematically visualized using NMDS (non-metric multidimensional scaling). The results (Figure 4) showed that the samples clustered by developmental stage, with significant separation between first instar nymphs and eggs, and between fifth instar nymphs and male and female adults. Stress = 0.0449 (*p* < 0.05), indicating that the NMDS analysis was well representative. The results of the PERMANOVA analysis (Figure 5) showed that the structure of intestinal bacterial flora in different developmental stages of *L. pratensis* was significantly different (R^2^ = 0.647, *p* = 0.007).

### 3.4. Functional Prediction of Intestinal Bacteria in L. pratensis

**Figure 6:**
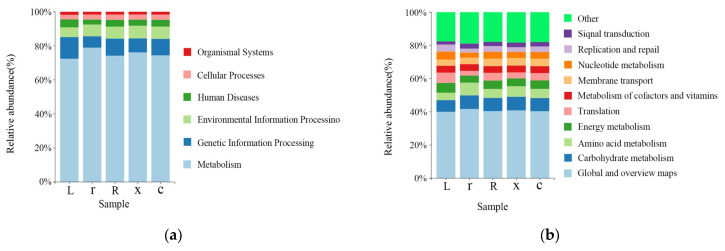
Analysis of Gut Bacterial KEGG Metabolic Pathways at Different Developmental Stages of *L. pratensis*: (**a**) Abundance at the first level of KEGG pathways; and (**b**) abundance at the second level of KEGG pathways.

The KEGG (Kyoto Encyclopedia of Genes and Genomes) functional prediction analysis showed that at the first-level functional level (Figure 6a), metabolism (75.21%), genetic information processing (9.46%), environmental information processing (6.88%), human diseases (3.82%), cellular processes (2.95%), organismal systems (1.69%) and genetic information processing (9.46%) were the dominant functional groups. The prediction showed that the abundance of metabolic pathways accounted for the largest share of the abundance of all first-order pathways, suggesting that the gut microbiota of *L. pratensis* primarily metabolizes various substances. Functional prediction showed that at the secondary level of the KEGG metabolic pathway (Figure 6b), there was no significant difference in the secondary metabolic pathways involved in gut microbiota between samples. The gut bacteria of *L. pratensis* at different developmental stages were mainly involved in global and overview maps (40.67%), carbohydrate metabolism (7.88%), amino acid metabolism (5.89%), and energy metabolism (4.98%) at the secondary classification level. It is predicted that gut bacteria play an important role in host metabolism. However, gene function predictions cannot fully reflect the actual functions of the intestinal bacterial population in *L. pratensis*, and these predicted functions need further analysis and verification.

## 4. Discussion

*L. pratensis* has the characteristics of wide host range, strong concealment of feeding and rapid population growth, and has become one of the important pests on cotton crops in Xinjiang. In this study, a total of 3378 ASVs were obtained based on high-throughput sequencing, belonging to 16 phyla, 25 classes, 54 orders, 85 families, 133 genera, and 187 species. Among them, we found that at the phylum level, Proteobacteria in the intestinal tract of *L. pratensis* accounted for a large proportion, making it the dominant phylum. In contrast, other phyla accounted for a small proportion. The high-abundance distribution of Proteobacteria was consistent with the general characteristics of insect gut microbiota and was closely related to the host’s key physiological functions. Proteobacteria are implicated in regulating growth, development, predatory capacity, and host fitness [21,22], findings consistent with those in other Hemipteran mirids, including *Adelphocoris suturalis* and *Apolygus lucorum* [23,24]. Intestinal microbial community composition varied across developmental stages of *L. pratensis*, as observed in previous studies. Zhu et al. [25] documented significant differences in gut bacterial community composition among developmental stages, with bacterial richness declining and diversity increasing from young nymphs to old nymphs and adults. Xue et al. [23] also reported higher gut bacterial richness in nymphs than adults, alongside higher diversity in adults. In this study, first instar nymphs of *L. pratensis* exhibited the highest gut bacterial richness and diversity across all developmental stages (from egg to adult). This result aligns with Xue et al. [26], who found the highest bacterial community diversity and richness in first and second instar nymphs of *Apolygus lucorum* (Hemiptera: Miridae), which was significantly higher than that in other developmental stages of *L. pratensis*. Wang et al. [27] reported the lowest gut microbial diversity in fifth instar nymphs and the highest in adults of *Nilaparvata lugens* (Hemiptera: Delphacidae), consistent with An et al. [24], who found lower microbial richness and diversity in nymphs than adults of the studied mirid. This consistency reflects a metabolic adaptation of the microbial community to gut environmental changes induced by dietary shifts during insect development [28]. Zhang et al. [29] noted that nymphs depend on high-nutrient host plants to shorten development and have low environmental tolerance, and that host plant selection determines their survival to adulthood. Liang [30] demonstrated that *L. pratensis* preys specifically on *Aphis gossypii* (Hemiptera: Aphididae), sharing omnivorous habits (herbivory and carnivory [31]) with other Lygus species but exhibiting a strong preference for plant-based food over predation [32]. In contrast to Li et al. [33], who identified *Enterococcus faecalis* as the dominant bacterium followed by *Lactococcus*, *Serratia*, and *Providencia*, the dominant floral composition in this study differed slightly. This may be related to the feeding habits, living environment, and interaction with other organisms. Therefore, the changes in intestinal bacterial populations across the different developmental stages of *L. pratensis* in this study may reflect gradual adaptation to new food sources and provide a new theoretical basis for further revealing the succession of gut microbiota.

In addition, the dominant intestinal bacteria of *L. pratensis* varied with developmental stage, with several genera exhibiting stage-specific abundance: *Acinetobacter* was predominant in first instar nymphs. At the same time, *Serratia* was abundant in fifth instar nymphs and in both female and male adults. The stage-specific presence and dynamic changes of these genera may be linked to the physiological metabolism of *L. pratensis*, facilitating its adaptation to diverse food sources and ecological niches across different developmental stages. Note that 16S rRNA gene sequencing (V3–V4) has limited resolution for species-level annotation, warranting further validation. Previous studies have demonstrated that *Serratia* synthesizes vitamins and amino acids and aids hosts in degrading cellulose, monoterpenes and diterpenes [34,35]; Proteobacteria promotes the oviposition behavior of female adults [36]; *Acinetobacter* enhances larval adaptability and mitigates inflammatory responses [37]; and *Lactobacillus* alleviates organophosphate pesticide-induced toxic damage in *Drosophila melanogaster* [38]. Collectively, these findings confirm that the gut microbiota is essential for key physiological processes of *L. pratensis*, including growth and development, environmental adaptation, and immune defense, and provide a novel theoretical foundation for the development of green prevention and control strategies against this pest.

PICRUSt functional prediction of the gut microbiota of *L. pratensis* across developmental stages revealed no significant interstage functional divergence but high metabolic activity in the intestinal bacterial community. As a core component of the host digestive system, these microbiota were primarily enriched in metabolic pathways for carbohydrates, amino acids, and energy, highlighting their critical roles in food digestion, nutrient absorption, and supply for *L. pratensis*. While the present study only infers the potential metabolic capacity of the gut bacterial community via gene functional annotation of sequencing data, with inherent discrepancies from the actual physiological functions of the flora in the host gut, it provides a vital theoretical basis for the targeted screening of intestinal bacterial genera and metabolic pathways of *L. pratensis*, as well as the development of green management strategies for this pest based on bacterial community regulation. This work thus warrants further systematic and in-depth research with targeted validation, including investigations into the transmission mode of the bacterial community across *L. pratensis* developmental stages, the response mechanism of the microbiota to host dietary shifts, and the regulatory effects of the bacterial community on host growth and reproduction. Such follow-up studies will provide novel theoretical support for establishing a comprehensive green management system for *L. pratensis*.

## 5. Conclusions

There were significant differences in the structural composition of the intestinal bacterial population of *L. pratensis* across developmental stages. The bacterial population diversity of first instar nymphs was the highest, and with age, it showed a dynamic change pattern, increasing first and then decreasing. The distribution of some endemic genera is related to specific developmental stages. Among them, *Acinetobacter* mainly exists in the first instar nymph stage, and *Serratia* is distributed in the fifth instar nymph and female and male adults. Functional prediction indicates that the intestinal bacterial population has a highly active metabolic potential, with the core involved in carbohydrate, amino acid, and energy metabolism. This discovery not only provides a new target for green prevention and control of *L. pratensis*, but also provides a theoretical basis for further elucidating the succession law and functional mechanism of its gut microbiota.

## Figures and Tables

**Table 1:** Basic information of 16S rRNA sequencing of gut bacteria at different developmental stages of *L. pratensis*.

Sample ID	Raw Reads	Effective Reads	Effective Ratio (%)	Average Length (bp)	ASVs	Coverage Index
L1	77,157	69,910	90.61	406	240	0.9999
L2	80,015	70,463	88.06	406	123	1.0000
L3	73,716	66,132	90.00	409	433	0.9999
r1	80,053	66,159	82.64	432	296	0.9999
r2	80,076	68,929	86.08	425	605	0.9999
r3	80,040	72,047	90.01	430	368	1.0000
R1	79,942	69,564	86.90	420	193	0.9999
R2	80,158	71,353	89.01	432	537	0.9999
R3	80,019	70,303	87.86	406	190	0.9999
c1	42,085	38,627	91.78	437	182	0.9999
c2	53,775	49,143	91.39	415	205	1.0000
c3	79,959	72,648	90.86	415	126	1.0000
x1	62,247	56,345	90.52	427	315	0.9998
x2	52,720	47,503	90.10	429	380	0.9999
x3	80,034	71,489	89.32	420	163	0.9999
Total	1,081,996	960,615	—	421	3378	—

L1~L3: Eggs; r1~r3: 1st instar nymphs; R1~R3: 5th instar nymphs; c1~c3: adult females; x1~x3: adult males.

## Data Availability

The original contributions presented in this study are included in the article. Further inquiries can be directed to the corresponding authors.
